# Large scale production of indole-3-acetic acid and evaluation of the inhibitory effect of indole-3-acetic acid on weed growth

**DOI:** 10.1038/s41598-021-92305-w

**Published:** 2021-06-22

**Authors:** Sakaoduoen Bunsangiam, Nutnaree Thongpae, Savitree Limtong, Nantana Srisuk

**Affiliations:** 1 Department of Microbiology, Faculty of Science, Kasetsart University, Chatuchak, Bangkok, 10900 Thailand; 2 Academy of Science, Royal Society of Thailand, Bangkok, 10300 Thailand

**Keywords:** Microbiology, Applied microbiology, Biotechnology

## Abstract

Indole-3-acetic acid (IAA) is the most common plant hormone of the auxin class and regulates various plant growth processes. The present study investigated IAA production by the basidiomycetous yeast *Rhodosporidiobolus fluvialis* DMKU-CP293 using the one-factor-at-a-time (OFAT) method and response surface methodology (RSM). IAA production was optimized in shake-flask culture using a cost-effective medium containing 4.5% crude glycerol, 2% CSL and 0.55% feed-grade l-tryptophan. The optimized medium resulted in a 3.3-fold improvement in IAA production and a 3.6-fold reduction in cost compared with those obtained with a non-optimized medium. Production was then scaled up to a 15-L bioreactor and to a pilot-scale (100-L) bioreactor based on the constant impeller tip speed (V_tip_) strategy. By doing so, IAA was successfully produced at a concentration of 3569.32 mg/L at the pilot scale. To the best of our knowledge, this is the first report of pilot-scale IAA production by microorganisms. In addition, we evaluated the effect of crude IAA on weed growth. The results showed that weed (*Cyperus rotundus* L.) growth could be inhibited by 50 mg/L of crude IAA. IAA therefore has the potential to be developed as a herbicidal bioproduct to replace the chemical herbicides that have been banned in various countries, including Thailand.

## Introduction

The global population and the corresponding food demand have been increasing annually in the past decades, and improvements in crop production are therefore needed. There is a high risk of crop yield loss, approximately 34% of which per year is caused by weeds^1,2^. Synthetic herbicides are widely used to control weeds, but their intensive use causes environmental pollution, the accumulation of harmful residues in soil and water resources, mammalian toxicity, and the evolution of herbicide resistance^3,4^. Due to human health concerns, hazardous herbicides such as paraquat, glyphosate and chlorpyrifos have already been banned in many countries worldwide. To minimize the use of toxic chemical herbicides, weed control using bioproducts called bioherbicides^5^ produced by eco-friendly technology will provide great benefits to both farmers and consumers.

Indole-3-acetic acid (IAA) is the most common auxin-class phytohormone and plays vital roles in plant growth and plant development processes, such as cell division, cell expansion, cell differentiation and fruit development^6,7^. IAA homeostasis is important for maintaining the hormonal balance at an optimum level suitable for normal plant growth and development. However, high levels of IAA can exert an inhibitory effect on plant physiological processes^8^. Several studies have reported that IAA at high concentrations inhibits seed germination and plant growth^9–12^. The inhibition scenario is caused by ethylene production due to aminocyclopropane-1-carboxylic acid synthase (ACC synthase) activity stimulated by high levels of auxin accumulation, which results in an ethylene burst that causes plant growth abnormalities and senescence. A more important factor implicated in growth inhibition and the actual phytotoxic response to auxins is the overproduction of abscisic acid (ABA)^13^. This is why IAA is naturally synthesized at low levels in plants or derived from plant-associated microbes.

Various microorganisms have shown the ability to produce IAA, such as bacteria, actinomycetes, fungi or yeast^14–21^. To produce safe agricultural products and reduce the use of chemical herbicides, potential IAA-producing microbes are attractive for use as producers for further commercial-scale IAA production. Yeast has been suggested to be a potent biotechnological microorganism that utilizes various substrates for growth and metabolite production. Our previous research showed that the basidiomycetous yeast *Rhodosporidiobolus fluvialis* DMKU-CP293 was a strong IAA producer^22^. However, little information on the optimization of yeast IAA production has been reported^23,24^. It is therefore of interest to optimize the IAA production medium for *R. fluvialis* DMKU-CP293. In biotechnology processes, the medium composition is of critical importance due to its impact on product concentration and production cost. In addition to the medium composition, which includes carbon sources, nitrogen sources, growth factors and l-tryptophan (a precursor of IAA biosynthesis), pH and temperature also affect microbial IAA production^15,24–26^. The optimization strategies employed in the present work were the one-factor-at-a-time (OFAT) and response surface methodology (RSM) approaches. OFAT, a traditional strategy to economically optimize bioproduct production, involves varying one factor while keeping the other factors constant. This approach is the simplest to implement and primarily helps in the selection of significant parameters affecting IAA yield. Alternatively, a statistical strategy for the simultaneous study of several factors influencing the production process at the same time, such as factorial experimental design and RSM, can be used.

Microbial IAA production may be a white biotechnology that could be employed to replace chemical IAA synthesis, which is expensive, unstable, and performed under extreme conditions using highly toxic substances^27^. Although microbial IAA production has been reported over the last decade, knowledge about large-scale IAA production in bioreactors is still lacking. Generally, prior to scaling up a bioprocess, the parameters are initially optimized through small-scale batch cultivations. However, those obtained parameters might not be transferable directly from small batch to large-scale fermentations. Therefore, an optimization process in a large-scale bioreactor is necessary to achieve high-yield IAA production. In this work, we aim to identify the optimal cost-effective medium and conditions for the pilot-scale production of IAA by the phylloplane yeast *R. fluvialis* DMKU-CP293 using OFAT and RSM. Furthermore, we also assess the efficacy of yeast IAA on weed suppression. We believe this study provides a reasonable bioprocess development approach for realizing the production of bioproducts to replace toxic substances for use in the agricultural sector.

## Results

### Optimization of IAA production by *Rhodosporidiobolus fluvialis* DMKU-CP293

#### One-factor-at-a-time (OFAT)

**Figure 1:**
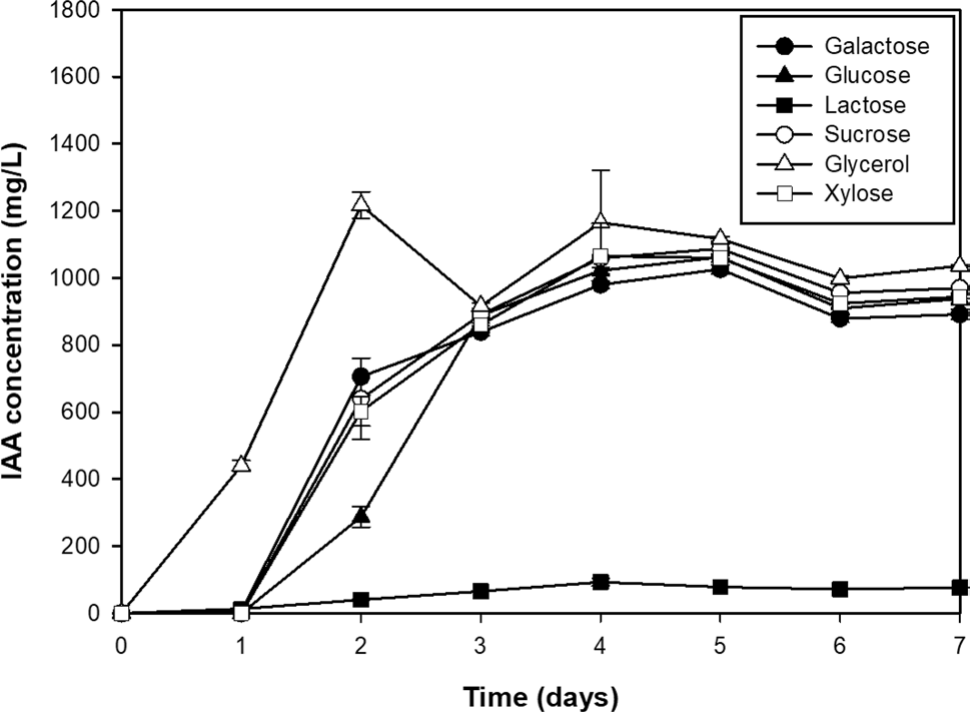
IAA production with various carbon sources by *R. fluvialis* DMKU-CP293 using YPD base medium (2% carbon source, 2% peptone, 1% yeast extract and 0.1% l-tryptophan) in shake-flask culture at 30 °C and 200 rpm for 7 days.

**Figure 2:**
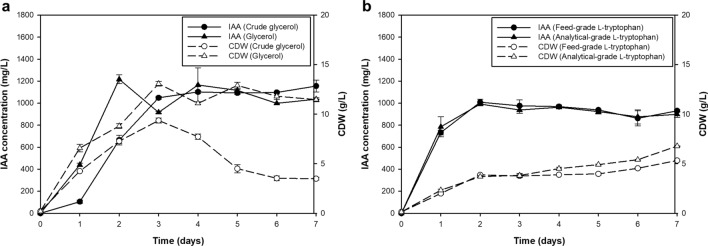
IAA production and cell dry weight of *R. fluvialis* DMKU-CP293 cultivated in a production medium containing (**a**) 2% pure glycerol or crude glycerol, 2% peptone, 1% yeast extract and (**b**) 0.1% analytical-grade l-tryptophan or feed-grade l-tryptophan in shake-flask culture at 30 °C and 200 rpm for 7 days.

When grown in the base medium (YPD broth) supplemented with 0.1% l-tryptophan, *R. fluvialis* DMKU-CP293 produced the maximum IAA level (1061.97 ± 4.29 mg/L) after 5 days of incubation (Fig. 1). To achieve maximum production, a one-factor-at-a-time (OFAT) approach was first applied. To select an appropriate carbon source, glucose was replaced by galactose, glycerol, lactose, sucrose, and xylose in turn. The highest IAA production (1217.25 ± 39.66 mg/L) was observed after 2 days of cultivation when glycerol was used (Fig. 1). To achieve low-cost IAA production, crude glycerol, which is a major byproduct of the biodiesel production process, was chosen as an alternative low-cost substrate to replace laboratory-grade glycerol. Fermentation performed with crude glycerol showed a similar level of IAA production with only one additional day of incubation (1048.91 ± 12.69 mg/L at 3 days of cultivation) compared with that required for pure glycerol (Fig. 2a). Crude glycerol was thus used in the subsequent studies.

To further reduce fermentation costs, a low-cost medium component was screened, and feed-grade l-tryptophan was used to replace analytical-grade l-tryptophan at the same concentration (0.1%). Figure 2b shows that a similar IAA concentration was obtained when the medium was supplemented with feed-grade l-tryptophan and analytical-grade l-tryptophan. Low-cost feed-grade l-tryptophan was therefore used in subsequent studies. The effect of temperature on IAA production was preliminarily studied between 30 and 35 °C. The results showed that *R. fluvialis* DMKU-CP293 produced a similar level of IAA production between 30 and 34 °C, but a significant decrease (*p* < 0.05) in IAA production was observed at 35 °C (data not shown). Therefore, subsequent IAA production was performed at 34 °C.

**Figure 3:**
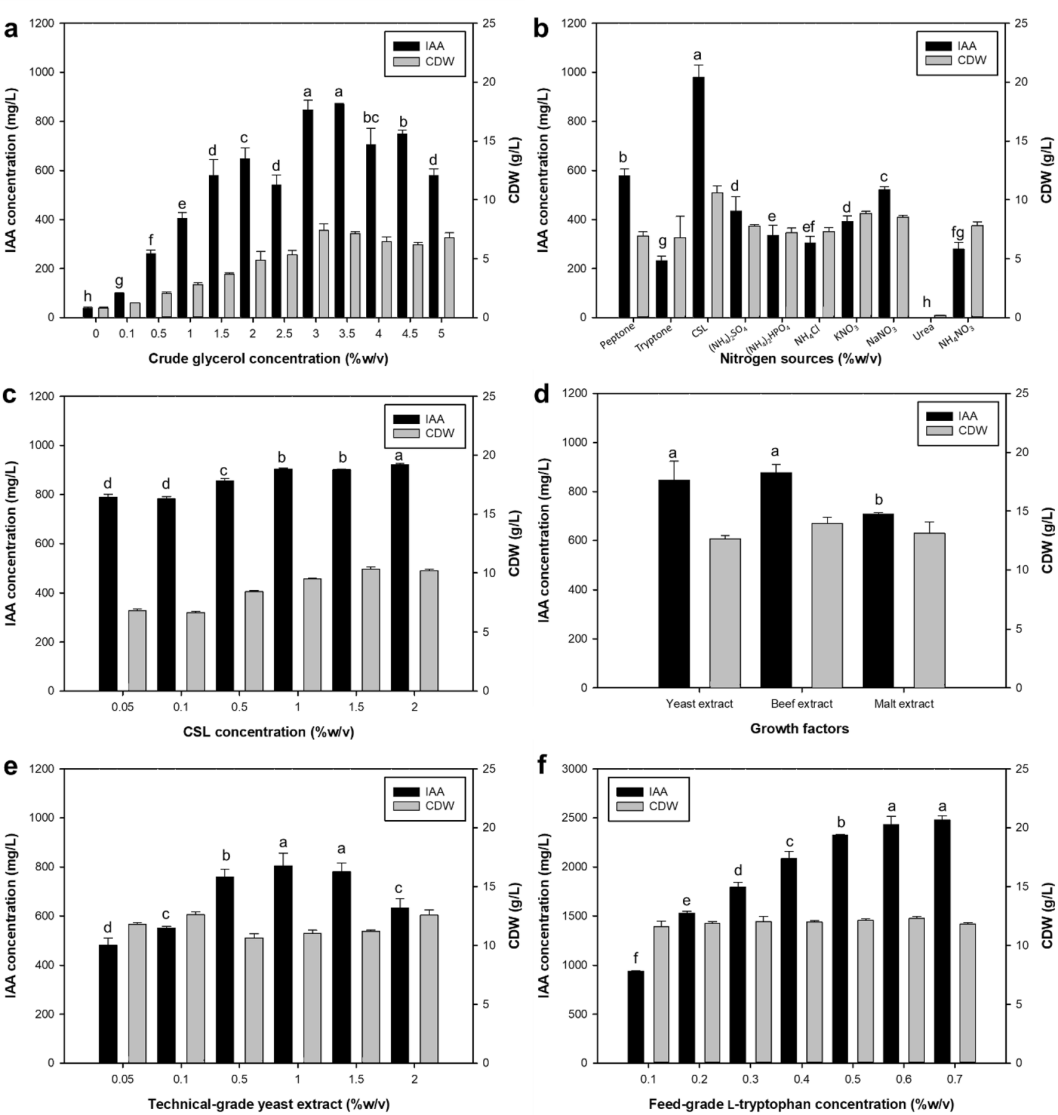
IAA production analyzed using the OFAT approach in media with varying parameters: (**a**) crude glycerol concentration, (**b**) nitrogen source, (**c**) CSL concentration, (**d**) growth factors, (**e**) technical-grade yeast extract concentration and (**f**) feed-grade l-tryptophan concentration at 34 °C and 200 rpm for 5 days. Different letters over the bars indicate significant differences between treatments using Duncan’s multiple range test (*p* < 0.05).

The effect of the crude glycerol concentration was studied within a range of 0 to 5% (w/v). The optimal IAA concentration was detected at 871.11 ± 1.84 mg/L, when 3.5% crude glycerol was used. No significant difference (*p* < 0.05) in IAA production was found when 3% and 3.5% crude glycerol were added to the production medium (Fig. 3a). A crude glycerol concentration of 3% was therefore selected for IAA production by *R. fluvialis* DMKU-CP293. Figure 3b indicates the results of the nitrogen source optimization experiment. NH_4_Cl, (NH_4_)_2_SO_4_, (NH_4_)_2_HPO_4_, KNO_3_, NaNO_3_, NH_4_NO_3_, peptone, tryptone, urea and corn steep liquor (CSL) were individually supplemented at 2% into the production medium containing 3% crude glycerol as the carbon source. The results showed that maximum IAA production (919.93 ± 6.30 mg/L) was achieved when 2% CSL was supplied as the nitrogen source under the conditions studied (Fig. 3c).

In addition to carbon and nitrogen sources (Fig. 3a–c), growth factors were also investigated (Fig. 3d). Technical-grade yeast extract was chosen for further study because it is less expensive than beef extract, and these two growth factors resulted in similar amounts of IAA production (875.78 ± 33.97 mg/L and 846.25 ± 77.24 mg/L when beef extract and technical-grade yeast extract were used, respectively). Figure 3e indicates that a high IAA concentration was obtained when the production medium was supplemented with 1% technical-grade yeast extract.

Tryptophan is generally considered a precursor for IAA production. In this study, the l-tryptophan concentration was therefore optimized. The results showed that IAA production increased from 939.84 ± 4.02 to 2432.61 ± 82.85 mg/L when the l-tryptophan concentration increased from 0.1 to 0.6%; however, the IAA concentration did not significantly (*p* < 0.05) increase when 0.7% l-tryptophan was used (2477.86 ± 42.34 mg/L) (Fig. 3f).

### Response surface methodology (RSM)

The important factors obtained from the preliminary OFAT screening were applied to the RSM approach. Four factors, crude glycerol (X_1_), CSL (X_2_), technical-grade yeast extract (X_3_), and feed-grade l-tryptophan (X_4_), were optimized through a central composite design (CCD), which is the most commonly used design for second-order models. CCD permits a lack-of-fit test to be performed from fewer experiments and also provides rotatability and orthogonality^28,29^. A total of 21 experiments were conducted to elucidate the effects of factors and their interactions on IAA production. A CCD simulation was carried out to predict the quadratic model that was most suitable to describe the relationship between the factors and responses. Regression was performed to fit the response function to the experimental data and resulted in models represented by the following Eq. (1):

1 $$\begin{gathered} {\text{IAA }}\left( {{\text{mg}}/{\text{L}}} \right){\text{ }} = {\text{ 1}}0{\text{81}}.{\text{6 }} - {\text{ 15}}0.{\text{86X}}_{{\text{1}}} - {\text{ 886}}.0{\text{2X}}_{{\text{2}}} - {\text{ 849}}.{\text{91X}}_{{\text{3}}} + {\text{ 3}}0.{\text{86X}}_{{\text{4}}} - {\text{ 41}}0.{\text{25X}}_{{\text{1}}} {\text{X}}_{{\text{2}}} + {\text{ 13}}.{\text{72X}}_{{\text{1}}} {\text{X}}_{{\text{3}}} - {\text{ 82}}0.{\text{25X}}_{{\text{1}}} {\text{X}}_{{\text{4}}} \hfill \\ - {\text{ 7}}.{\text{59X}}_{{\text{2}}} {\text{X}}_{{\text{3}}} - {\text{ 47}}.{\text{67X}}_{{\text{2}}} {\text{X}}_{{\text{4}}} - {\text{ 527}}.{\text{25X}}_{{\text{3}}} {\text{X}}_{{\text{4}}} + {\text{ 38}}.{\text{82X}}_{{\text{1}}} ^{{\text{2}}} + {\text{ 281}}.{\text{24X}}_{{\text{2}}} ^{{\text{2}}} + {\text{ 322}}.0{\text{6X}}_{{\text{3}}} ^{{\text{2}}} - {\text{ 443}}.{\text{47X}}_{{\text{4}}} ^{{\text{2}}} \hfill \\ \end{gathered}$$

where IAA (mg/L) represents the predicted IAA production response; X_1_ is the crude glycerol concentration, X_2_ is the CSL concentration, X_3_ is the technical-grade yeast extract concentration and X_4_ is the feed-grade l-tryptophan concentration.

**Figure 4:**
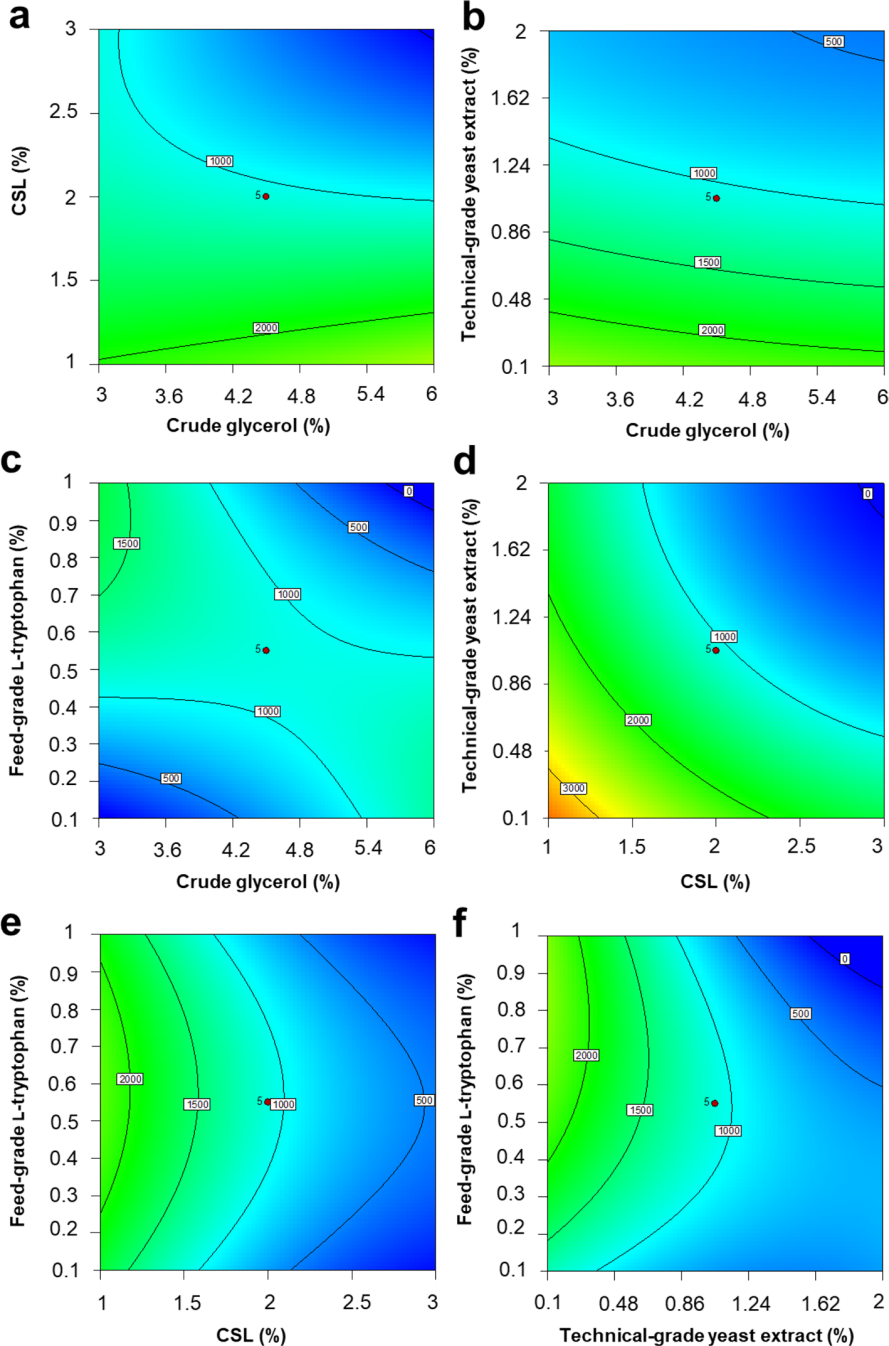
Contour plots of IAA production by the yeast *R. fluvialis* DMKU-CP293 using central composite design showing interactions between crude glycerol, CSL, technical-grade yeast extract, and feed-grade l-tryptophan after 5 days of incubation at 200 rpm and 34 °C.

The statistical analysis of factor significance was described by the analysis of variance (ANOVA) results shown in Table 1. The determination coefficient (*R*^*2*^), correlation, and model significance (*p* value) were used to analyze the fit of the model. The model obtained in this work showed a coefficient value (*R*^*2*^) of 0.9429 for IAA production, indicating that only 5.71% of the total variation was not explained by the model. The adjusted *R*^*2*^ was 0.8096, indicating good agreement between the obtained and predicted values for the output response. The model significance (*F*-value) indicating data variation around the mean was also measured. The probability value of the model (*p*-value prob > *F*) was less than 0.05, implying that the model could be considered to be significant. In addition, the probability value also indicated that the present model predicted the experimental results well. The optimum values of the variables were determined to maximize IAA production by *R. fluvialis* DMKU-CP293. From the regression model, contour plots assisted in understanding the effect as well as the interactions of the four factors, i.e., crude glycerol, CSL, technical-grade yeast extract and feed-grade l-tryptophan. Response surface plots were drawn to illustrate the pairwise combinations of the four variables (Fig. 4). According to Fig. 4a–f, high levels of crude glycerol and feed-grade l-tryptophan and low levels of CSL and technical-grade yeast extract enhanced IAA yield. A low level of technical-grade yeast extract was found to increase IAA production, as shown in Fig. 3e. However, the summary of the criteria set for the optimization run obtained from the CCD showed that yeast extract could be omitted from the culture because the optimized level of yeast extract provided was “− 0.55” (Fig. S1c).

**Table 1:** Regression analysis quadratic model of CCD.

Source	Sum of squares	df	Mean square	*F*-value	*p-*value Prob > *F*
Model	2.440E + 007	14	1.743E + 006	7.07	0.0121
X_1_-crude glycerol	1.287E + 005	1	1.287E + 005	0.52	0.4970
X_2_-CSL	4.441E + 006	1	4.441E + 006	18.02	0.0054
X_3_-technical-grade yeast extract	9.865E + 006	1	9.865E + 006	40.04	0.0007
X_4_-feed-grade l-tryptophan	5385.54	1	5385.54	0.022	0.8873
X_1_X_2_	5.577E + 005	1	5.577E + 005	2.26	0.1832
X_1_X_3_	1504.85	1	1504.85	6.108E − 003	0.9402
X_1_X_4_	2.230E + 006	1	2.230E + 006	9.05	0.0238
X_2_X_3_	461.11	1	461.11	1.871E − 003	0.9669
X_2_X_4_	7528.89	1	7528.89	0.031	0.8670
X_3_X_4_	2.224E + 006	1	2.224E + 006	9.03	0.0239
X_1_^2^	22,521.91	1	22,521.91	0.091	0.7726
X_2_^2^	1.182E + 006	1	1.182E + 006	4.80	0.0710
X_3_^2^	1.550E + 006	1	1.550E + 006	6.29	0.0460
X_4_^2^	2.939E + 006	1	2.939E + 006	11.93	0.0136
Residual	1.478E + 006	6	2.464E + 005		
Lack of fit	8.030E + 005	2	4.015E + 005	2.38	0.2087
Pure error	6.753E + 005	4	1.688E + 005		

*R*^*2*^ = 0.9429, Adjusted *R*^*2*^ = 0.8096.

To confirm the ability of the model to predict the maximum response, triplicate sets of experiments were performed using the optimized medium composition, 4.5% crude glycerol, 2% CSL and 0.55% feed-grade l-tryptophan, with incubation at 34 °C for 5 days on an orbital shaker at 200 rpm. These sets of conditions were also used to validate and predict the responses using the model equation. The observed IAA concentration (3514.44 ± 52.37 mg/L) was close to the predicted value (3474.36 mg/L), confirming the validity and adequacy of the model. Using the optimized medium, IAA production was increased up to 3.3-fold compared with that obtained using unoptimized medium. In addition, IAA productivity and IAA yield at the end of 5 days of fermentation were 29.29 mg/L/h and 0.65 mg IAA/mg l-tryptophan, respectively.

### IAA production in the pilot-scale 100-L bioreactor

**Figure 5:**
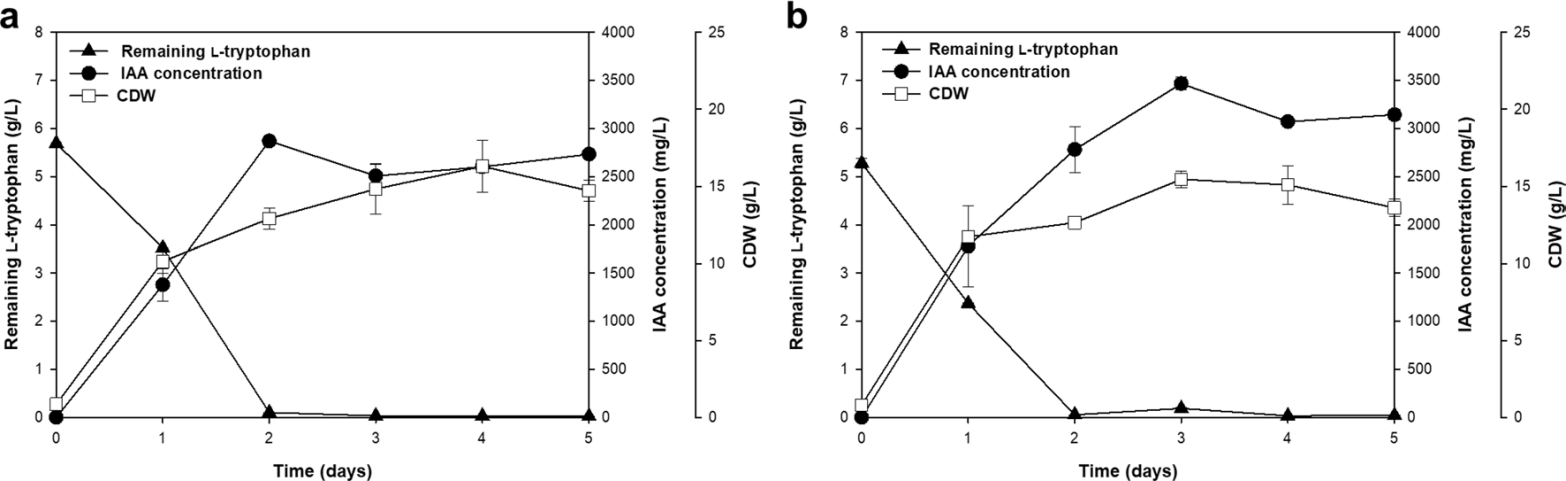
The IAA concentration (mg/L), remaining l-tryptophan and cell dry weight in the batch fermentation of *R. fluvialis* DMKU-CP293 in (**a**) 2-L and (**b**) 15-L stirred tank bioreactors using the optimal IAA production medium at 34 °C, constant agitation speed (400 rpm), 10% inoculum size and 1 vvm aeration.

**Figure 6:**
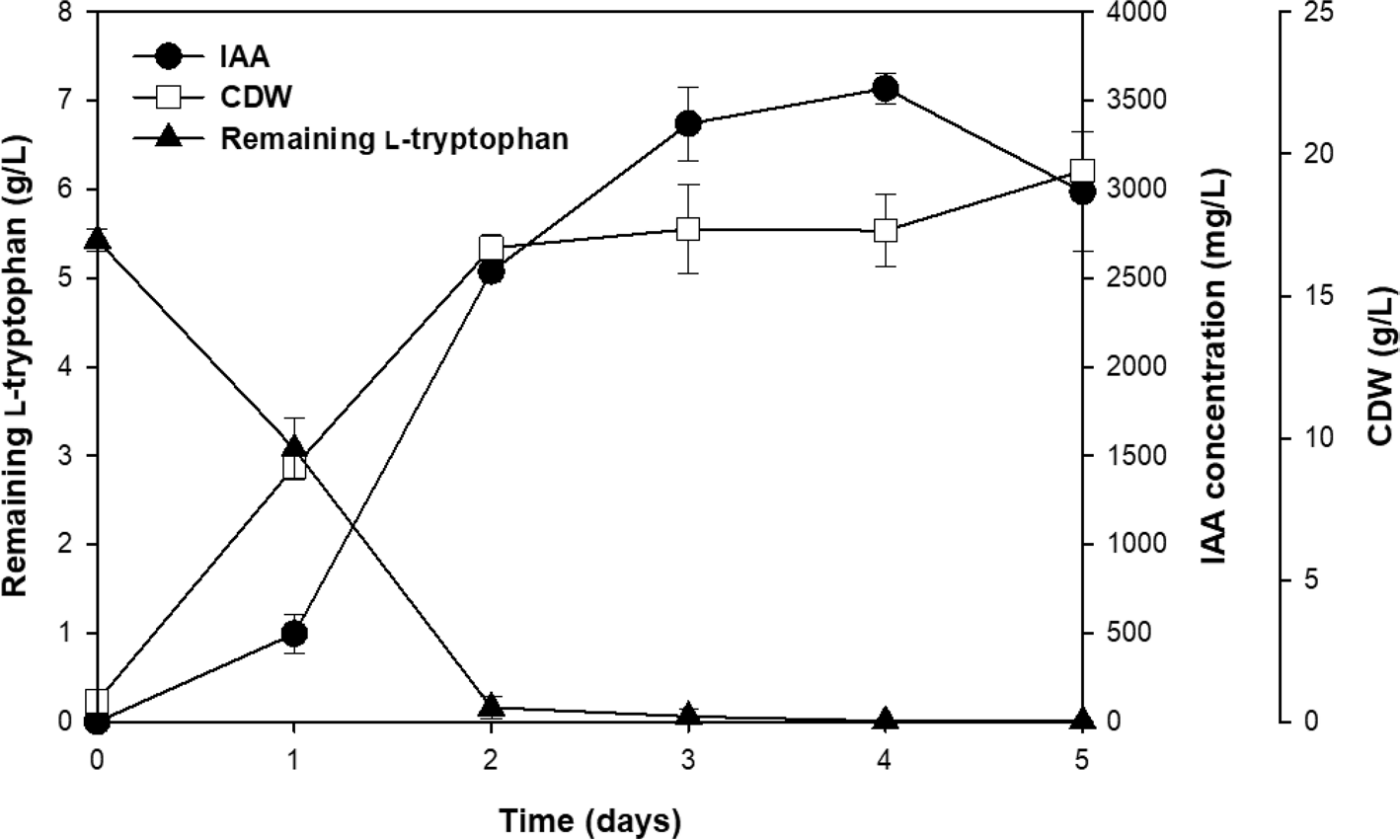
IAA production, l-tryptophan consumption and cell dry weight in batch fermentation by *R. fluvialis* DMKU-CP293 at the 100-L pilot scale at 34 °C, constant agitation speed (170 rpm), 10% inoculum size, and 1 vvm aeration.

To achieve high-level IAA production in the bioreactor, the inoculum size and agitation speed were preliminarily investigated in a 2-L laboratory-scale bioreactor. The inoculum size (5–20%) and agitation speed (200 rpm, 300 rpm, and 400 rpm) were varied, and the aeration rate was fixed at 1 vvm. The optimal conditions were 10% inoculum size, 400 rpm agitation and 1 vvm aeration (data not shown). The highest IAA concentration, 2870.15 ± 11.38 mg/L (equivalent to an IAA yield of 0.51 mg IAA/mg l-tryptophan), was achieved after 2 days at 34 °C (Fig. 5a). IAA production was scaled up into a 15-L bioreactor with the same cultivation medium and conditions used in the 2-L bioreactor, and the maximum IAA level, 3468.17 ± 66.61 mg/L (equivalent to an IAA yield of 0.68 mg IAA/mg l-tryptophan), was obtained after 3 days (Fig. 5b). To further scale up IAA production, a 100-L pilot-scale bioreactor was set up with a constant impeller tip speed (V_tip_). Since the agitation rate was scaled based on the V_tip_ (proportional to ND_I_, where N is the agitation speed and D_I_ is the impeller diameter), it was expected that the broth viscosity would remain similar. The results showed that IAA concentrations reached 3569.32 ± 85.28 mg/L after 4 days, corresponding to an IAA yield of 0.66 mg IAA/mg l-tryptophan (Fig. 6), when the V_tip_ value was kept constant at 6.96 m/s (corresponding to an agitation speed of 170 rpm).

Cost efficiency is always a key factor in the industrialization of products. An increase in IAA productivity will obviously reduce the overall production cost and, hence, the cost of the product. This study showed that the highest IAA production was obtained when low-cost substrates, i.e., crude glycerol, CSL and feed-grade l-tryptophan, were used. The production cost per liter of the optimal medium obtained via the RSM approach was 2.10 USD/L, while the original medium (YPD broth) cost 7.61 USD/L, as shown in Table 2. This means that IAA production by *R. fluvialis* DMKU-CP293 in the optimal medium provided an effective cost reduction of 3.6-fold. Based on the production scale of the bioreactors, 2-L, 15-L and 100-L production cost 0.74, 0.60 and 0.60 USD/g IAA, respectively.

**Table 2:** Cost of IAA production medium in 100 L bioreactor.

Production medium	Production cost per L (USD)	Obtained IAA (mg IAA/L)
YPD broth + 0.1% analytical-grade l-tryptophan (shaking flask)	7.61	1061.97
Optimized IAA production medium	2.10	3569.32

### Evaluation of the inhibitory effect of IAA on weed growth

**Figure 7:**
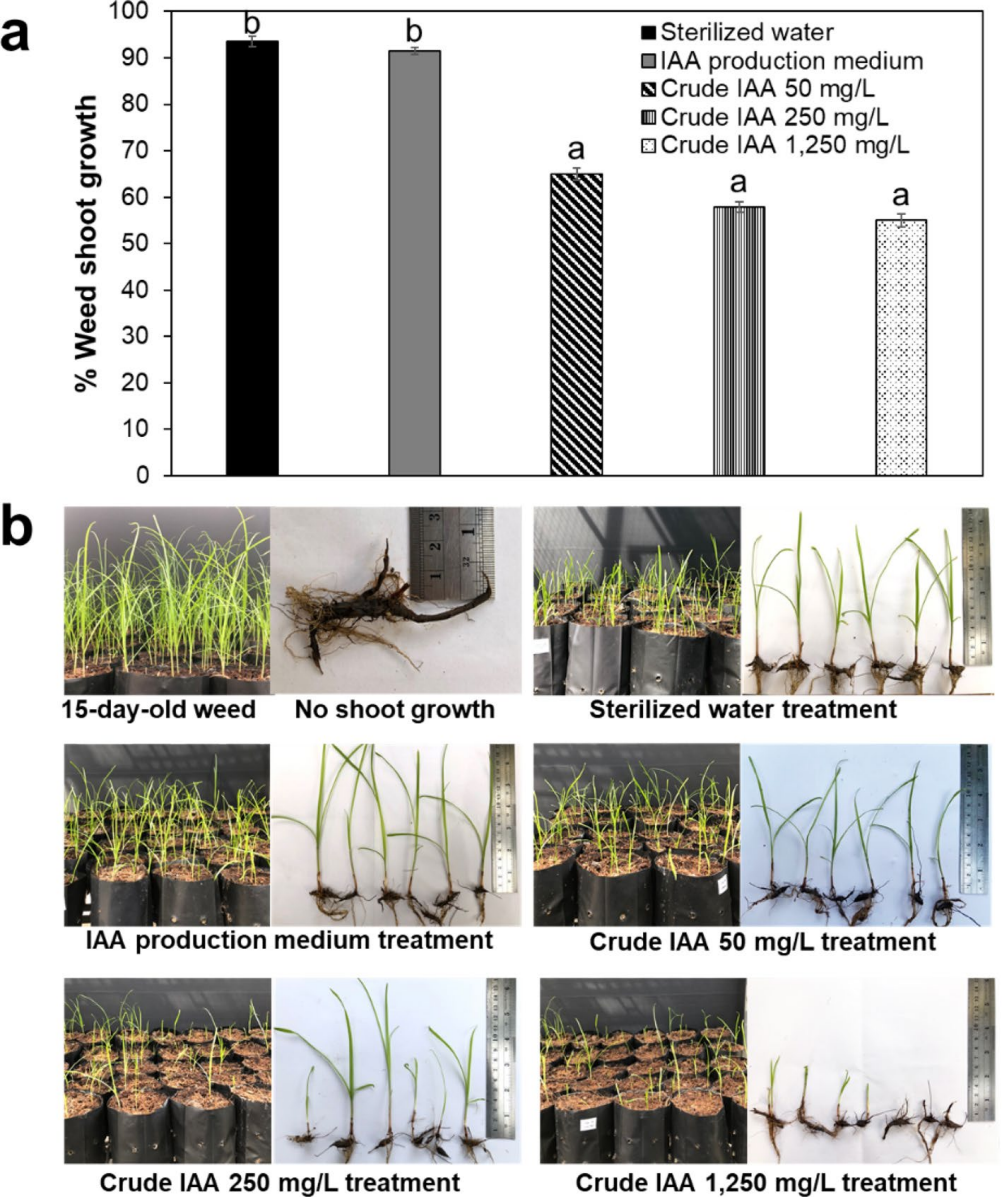
Effect of (**a**) crude IAA produced by *R. fluvialis* DMKU-CP293 on weed shoot growth and (**b**) weed growth in plastic planting bags in control and crude IAA treatments after 9 days in a greenhouse. Different letters above the bars indicate significant differences between treatments using Duncan’s multiple range test (*p* < 0.05).

This part of the study aimed to evaluate the effect of IAA on weed growth inhibition. Nutgrass (*Cyperus rotundus* L.) tubers were grown for 15 days in a plastic planting bag with sterile soil to test weed germination. To imitate farmland management practices prior to the planting of a main crop, 15-day-old weed shoots were discarded, and the remaining underground weed tubers were treated with three concentrations of crude IAA (50, 250 or 1250 mg/L), the IAA production medium or sterile distilled water (control). After 9 days of treatment, weed shoot growth was recorded. The growth of weeds treated with the IAA production medium was not significantly different (*p* < 0.05) from that of weeds treated with the control (Fig. 7a). This result indicates that the IAA production medium had no effect on weed growth. Crude IAA significantly suppressed (*p* < 0.05) weed shoot growth compared with the control and medium treatments. At 50, 250 and 1250 mg/L of crude IAA, weed shoot growth was 65, 57.86 and 55% of that in the control, indicating inhibitory effects of 30.53, 38.17 and 41.22%, respectively (Fig. 7a). Weed shoot length was evidently reduced by 1250 mg/L crude IAA compared to that in the water and IAA production medium treatments (Fig. 7b). Overall, the results suggest that crude IAA at least 50 mg/L may suppress nutgrass growth.

## Discussion

The phylloplane is the surface or aboveground parts of plants and has been recognized as an important habitat for microorganisms^30^. Many phylloplane yeasts have shown the ability to synthesize plant developmental hormones^14,24,31–33^. In the present study, the corn phylloplane yeast *R. fluvialis* DMKU-CP293 produced the highest amount of IAA, 3514.44 mg/L, when cultured in an IAA production medium containing crude glycerol, CSL and feed-grade l-tryptophan as the carbon, nitrogen, and IAA precursor sources, respectively, in a shaking flask. Low-cost substrates were used in this work. The impurities contained in crude glycerol (methanol, NaOH, esters and sulfur compounds) may affect yeast growth; however, crude glycerol and pure glycerol promoted similar levels of IAA production by *R. fluvialis* DMKU-CP293. This is probably due to the residual proteins and minerals^34^ in crude glycerol that may support yeast IAA production. Feed-grade l-tryptophan, which was used as a precursor for IAA synthesis, played a major role in reducing IAA production costs, as reported in Nutaratat and Srisuk^35^. In addition, *R. fluvialis* DMKU-CP293 showed a high IAA yield when CSL was provided in the production medium as a nitrogen source. This result is consistent with that of Nutaratat, et al.^24^, who indicated that *Rhodosporidium paludigenum* DMKU-RP301 also exhibited peak IAA production (314.8 mg/L) when CSL was used as a nitrogen source. CSL is a major byproduct of cornstarch processing and is a low-cost source of proteins, amino acids, minerals and vitamins. It has been reported to be a potential nitrogen source for the production of bioproducts^36–39^. The optimized medium developed in this study (containing crude glycerol, CSL, and feed-grade l-tryptophan) was more cost-effective than other media and achieved high IAA production with a less-expensive fermentation medium. Several low-cost substrates for IAA production have been studied, such as agroindustrial residues, agrowaste substrates, jatropha seedcake, and sweet whey, but the cost of using these substrates has not yet been estimated^40–44^. As shown in Table 2, the fermentation costs of using the initial and optimized medium in the 100-L bioreactor were compared. The total fermentation costs of IAA production medium per liter using the initial and optimized media were 7.61 USD and 2.10 USD, respectively. The low-cost medium developed for IAA production with *R. fluvialis* DMKU-CP293 may facilitate process optimization for economical IAA production at the industrial scale. Recently, high level (7 g/L) of IAA production by metabolically engineered *Escherichia coli* has been reported^45^. However, this engineered bacterium required inducer and selective pressure to maintain high IAA production. The protocol may not be suitable for industrial scale IAA production due to high cost is required to maintain bacterial IAA production stability.

*Rhodosporidiobolus fluvialis* DMKU-CP293 showed high IAA production within only 4 days of fermentation, which could minimize production costs in terms of short-term fermentation. Compared to the production periods in other studies, the yeast *R. fluvialis* DMKU-CP293 required a short IAA production period. Nutaratat, et al.^24^ reported high IAA levels generated by the yeast *Rh. paludigenum* DMKU-RP301 after 7 days of fermentation, whereas *Colletotrichum fructicola* CMU-A109 took 26 days to produce its maximum IAA concentration^16^. In addition to medium components, fermentation temperature is another parameter to consider. Our studies showed high IAA production at 34 °C, which is slightly higher than the ordinary yeast growth temperature. Applying this temperature would help to somewhat reduce cooling costs for IAA production in tropical countries, including Thailand.

In the current study, IAA optimization was carried out by a combination of the OFAT and RSM approaches. The results from the medium optimized by RSM ultimately provided a high IAA yield that was 3.3-fold higher than that of the non-optimized medium. RSM is a statistical optimization method that has been successfully applied by many researchers to improve IAA production^21,24,44,46^. RSM allows more factors to be evaluated at the same time for their effects on IAA production than the OFAT approach and provides less variability in the experiments. In addition, the interactions between factors can be estimated systematically when using RSM, but they cannot be estimated by OFAT^47^. The results obtained in the present work are consistent with the report of Nutaratat, et al.^24^, which determined the final optimal conditions for IAA production using RSM after the OFAT approach. In addition, we also found that yeast extract did not enhance IAA production by this yeast, possibly because the CSL provided enough growth factors^48,49^ for IAA production.

High-quantity production is a key factor in successful industrial bioprocesses. Fermentation was scaled up to a 15-L bioreactor in this work on the basis of the optimum conditions identified in a 2-L bioreactor. The experiment aimed to obtain cell and product quantities at a large scale (the industrial or pilot-plant scale) with at least the same efficiency as that obtained at a laboratory scale. However, upscaling is not an easy task because different reduction foam efficiencies, substrate bioavailability levels, oxygen transfer efficiencies and adverse physical or biological effects can occur at different fermentation scales^50^.

For bioproduction processes involving aeration, the biomass yield and growth-associated products have been shown to decrease when performed at a large scale^51^. Our research revealed that batch-fermentation IAA production reached 3468.17 ± 66.61 mg/L in the 15-L reactor, representing a yield of 0.68 g IAA/g l-tryptophan, which is the highest production ever reported by yeast^52^. Complete l-tryptophan consumption by *R. fluvialis* DMKU-CP293 was shown after 2 days of fermentation, suggesting that fed-batch fermentation may help to avoid inefficiencies in the l-tryptophan supply and hence incomplete IAA production. Currently, Ozdal, et al.^53^ have demonstrated successful IAA production using immobilized *Arthrobacter agilis* cells with 13 times reuse in flask scale production (25 mL medium in 250 mL flask). Microbial immobilized cells have been shown to have many advantages such as higher cell density and stability, reduced reaction time, long term reuse, easy separation from the production medium, and higher product yield^54,55^. However, large scale IAA production using immobilized yeast cells have to be analyzed both in terms of growth condition and type of aeration. Fed-batch, repeated-batch and continuous fermentation using immobilized *R. fluvialis* DMKU-CP293 will therefore be of interest to further investigate.

Surprisingly, higher IAA production was found in the 15-L bioreactor than in the 2-L bioreactor. This may be due to differences in the bioreactor design, such as in the impeller spacing, baffle and sparger specifications, and vessel ratio, which helped to provide better yeast growth and IAA production in the 15-L bioreactor than in the 2-L bioreactor. In 2015, Shivanandappa, et al.^56^ reported that impeller position affected the growth yield of *Bordetella pertussis* strain 509 during large-scale batch fermentation. A single impeller was found to improve the growth yield of *B. pertussis* strain 509, whereas two and three impellers located at various positions resulted in a decrease in the growth rate due to the disturbance of vortex flows and broth mixing resulting in less dissolved oxygen being transferred.

Tip velocity (V_tip_) is another useful parameter for increasing the production size to the pilot or industrial scale^57–59^. V_tip_ was shown to be an effective parameter for IAA production by *R. fluvialis* DMKU-CP293 in a pilot-scale 100-L bioreactor. Based on the V_tip_ scaling-up strategy, this research revealed a similar level of IAA production in a 100-L bioreactor to those obtained in a shaking flask and a laboratory-scale 15-L bioreactor. This is the first report of microbial IAA production in a pilot-scale bioreactor using low-cost substrates, i.e., crude glycerol, CSL, and feed-grade l-tryptophan. All results suggested that the optimized medium obtained in this study could be used as a cost-effective medium for IAA production by *R. fluvialis* DMKU-CP293 at the industrial scale.

The development of biological products that can be used for eco-friendly agriculture is urgently required to reduce environmental pollution due to the current excessive use of harmful agrochemicals. IAA can have both positive and negative effects on plants depending on the dosage used and the plant species. The application of IAA at low levels (approximately 1 nmol/L–10 µg/L) was reported to promote plant growth^43,60^, but higher amounts of IAA showed adverse effects on plant growth^61^. Our research attempted to harness the adverse effects of IAA to control weed growth. *Cyperus rotundus* L., nutgrass, shows rapid growth and good tolerance to several stress conditions, including drought. This grass is truly difficult to remove from land and farms because its tubers remain underground after land development and prior to plantation. It usually grows along with or overtakes the growth of main crops due to its drought tolerance and recovers to grow immediately in the rainy season or after crop watering. This grass has therefore been considered to be the world's worst weed^62^. This grass can be killed or inhibited by chemical herbicides such as glyphosate and paraquat; the residues of these herbicides are well known to cause environmental pollution and are harmful to human health. The inhibitory effect of IAA could be exploited to allow its use as an alternative herbicide due to its negative effect on plant growth when applied at high concentrations. The application of high-load IAA as a bioherbicide does not affect crops since IAA degrades under high light intensities^63^. The results revealed that 50 mg/L showed an inhibitory effect on the growth of the weed *Cyperus rotundus* L. This is consistent with the report of Dahiya et al.^64^, which showed an inhibitory effect of bacterial IAA at 53.80 µg/mL on *Avena fatua* (wild oat). At a high IAA concentration (1250 mg/L), shoot growth was evident. This observation was consistent with the study of Cline^65^ reporting that 1% exogenous IAA (equivalent to 10000 mg/L) significantly inhibited lateral bud outgrowth in the tested plant species (*Ipomoea nil*, *Helianthus annuus*, *Lycopersicon esculentum* (VNF8), *Pisum sativum*). In another study, Kim and Krcmcr^66^ reported that *Bradyrhizobium japonicum* GD3 and *Pseudomonas putida* GD4 produced high IAA concentrations of 64 mM and 6.9 mM (equivalent to 11210 mg/L and 1210 mg/L, respectively) that significantly reduced the growth of morning glory (*Ipomoea* spp.). Research on bioherbicides should be attracting broad interest due to bans on certain agrochemicals and environmental concerns related to farmer health. Moreover, the development of product formulations with longer shelf lives is required for successful commercialization.

## Materials and methods

### Microorganism and cultivation medium

The corn phylloplane yeast *Rhodosporidiobolus fluvialis* DMKU-CP293 (LC379571) was grown on yeast extract peptone dextrose (YPD) agar (1% yeast extract, 2% peptone, 2% glucose and 1.5% agar). Yeast inoculum was cultivated in 50 mL of YPD broth and incubated on an orbital shaker (JS Research Inc., South Korea) at 170 rpm and 30 °C for 16–18 h. The yeast cells were collected by centrifugation for 5 min at 10000 × *g*, washed twice with sterilized distilled water, and transferred into a 250 mL Erlenmeyer flask containing 50 mL of YPD medium supplemented with 0.1% (w/v) l-tryptophan. The medium initial pH was adjusted to 6, and the initial optical density at 600 nm (OD_600_) was adjusted to 0.2 prior to incubation on an orbital shaker at 200 rpm and 30 °C. Samples were taken every 24 h. The culture broth was collected by centrifugation for 5 min at 10000 × *g,* and then the supernatant was analyzed for its IAA concentration by high-performance liquid chromatography (HPLC).

### Medium optimization in shake-flask cultivation

#### Experimental design for optimizing IAA production using low-cost substrates

##### One-factor-at-a-time (OFAT)

The OFAT approach was applied to preliminarily screen for influencing factors using YPD as the base medium. Various nutritional conditions were studied: different carbon sources (galactose, glucose, lactose, sucrose, glycerol, and xylose), crude glycerol concentrations (0–5%), nitrogen sources (NH_4_Cl, (NH_4_)_2_SO_4_, (NH_4_)_2_HPO_4_, KNO_3_, NaNO_3_, NH_4_NO_3_, peptone, tryptone, urea, CSL, CSL concentrations (0.05–2%), growth factor sources (yeast extract, malt extract, and beef extract), technical-grade yeast extract concentrations (0.05–2%) and feed-grade l-tryptophan concentrations (0.1–0.7%). High-purity glycerol (Ajax Finechem Pty Ltd., Australia), laboratory-grade yeast extract (Becton, Dickinson and Company, USA) and analytical-grade l-tryptophan (Acros Organics, USA) were used in the high-cost medium whereas crude glycerol (Global Green Chemicals Public Company Limited, Thailand), technical-grade yeast extract (Becton, Dickinson and Company, USA) and feed-grade l-tryptophan (Ajinomoto Animal Nutrition Europe, France) were used in the low-cost medium. The initial medium pH was adjusted to 6 in all experiments. The incubation times (0–7 days) and temperatures (30–35 °C) were also optimized. All experiments were performed in triplicate, and the results are reported as the mean of these replications.

##### Response surface methodology (RSM)

Central composite design (CCD)^67^ was used to identify the optimum conditions for IAA production after the preliminary range of variables was determined through OFAT. The CCD included an embedded factorial or fractional factorial matrix with center points and star points around the center point. The distance from the center of the design space to a factorial point was ± 1 unit for each factor, and the distance from the center of the design space to a star point was ± α, where |α|> 1. An axial distance (+ α) of 1.68 was chosen to make the design rotatable. The variables included crude glycerol, CSL, technical-grade yeast extract, and feed-grade l-tryptophan at five coded levels, − α, − 1, 0, + 1, + α, which are shown in Table S1. The CCD included 21 experimental trials, with 5 trials as replications of the center points. The results from the CCD were then statistically evaluated by Design Expert 10 software (Stat-Ease Inc., England). All experiments were performed in triplicate, and the results are reported as the mean of these replications.

### Batch cultivation of IAA using low-cost substrates in a laboratory-scale bioreactor

The effects of fermentation parameters such as agitation speed and inoculum size were examined to maximize IAA production using a bench scale 2-L bioreactor (BIOSTAT B, B. Braun Biotech International, Germany) with a 1.5-L working volume. Yeast inoculum was prepared in a 250 mL Erlenmeyer flask containing 50 mL YPD broth and then incubated on an orbital shaker (JS Research Inc., South Korea) at 170 rpm and 30 °C for 24 h. The culture OD_600_ reached approximately 4.0. The yeast inoculum was transferred to the IAA production medium obtained in the previous experiment (4.5% crude glycerol, 2% CSL and 0.55% feed-grade l-tryptophan). The process parameters included different agitation speeds (200, 300 and 400 rpm) and inoculum sizes (5%, 10%, 15%, and 20%), and the processes were performed in a 2-L bioreactor for maximum IAA production. The aeration and incubation temperatures were kept constant at 1.5 L/min (1 vvm) and 34 °C, respectively. The culture pH and dissolved oxygen percentage were monitored by pH electrodes (405-DPAS-SC-K8S/225, Mettler Toledo, Switzerland) and dissolved oxygen sensor probes (InPro 6800, Mettler Toledo, Switzerland), respectively.

A stainless steel 15-L stirred tank bioreactor (BIOSTAT C, B. Braun Biotech International, Germany) was used in the scaling-up of the fermentation. The fermentation conditions and optimized IAA production medium obtained from the 2-L bioreactor were applied in the 15-L bioreactor. The culture pH was measured offline by a benchtop pH meter equipped with a pH electrode LE438 (FiveEasy F20, Mettler Toledo, Switzerland). Samples were taken daily for 7 days and centrifuged (10000 × *g* for 5 min) to collect the supernatant prior to IAA analysis by HPLC. The experiment was performed twice.

### Pilot-scale IAA production in a 100-L bioreactor

Pilot-scale batch culture was carried out in a 100-L stirred tank model MPF-U2W (B.E. MARUBISHI Co., Ltd., Japan). The impeller tip velocity (V_tip_) was focused on as a scaling-up parameter and was calculated as described in Wang, et al.^68^ and Eq. (2):

2 $${\text{V}}_{{{\text{tip}}}} = {\text{ 2}}\pi {\text{ND}}_{{\text{I}}}$$

where V_tip_ is the impeller tip speed, N is the agitation speed, and D_I_ is the impeller diameter. The first seed culture was prepared in a 1000 mL Erlenmeyer flask containing 250 mL YPD broth in the same manner as previously described. For the scaled-up culture, 500 mL of the first seed culture was inoculated into a 15-L bioreactor containing 5.5 L of YPD medium and cultivated for 24 h at 30 °C at 200 rpm and 1 vvm. Six liters of the second seed culture obtained from the 15-L bioreactor was transferred to the 100-L bioreactor containing 54 L of production medium obtained from the previous experiment (4.5% crude glycerol, 2% CSL and 0.55% feed-grade l-tryptophan) and cultivated for 5 days at 34 °C with 1 vvm of aeration and 170 rpm of agitation. Samples were taken daily and centrifuged (10000 × *g* for 5 min), and the supernatant was collected and analyzed for its IAA concentration by HPLC. The culture pH was measured offline by a benchtop pH meter equipped with a LE438 pH electrode. The experiment was performed twice.

### Assessment of the inhibitory effect of IAA on weed growth

Tubers of *Cyperus rotundus* L. were obtained from Suan Kaset Insee, a local farm in Chonburi province, Thailand by personal contact prior to discarding them as weed. Seven tubers of *Cyperus rotundus* L. were grown in plastic planting bags (size: 5 in. × 10 in.) filled with sterilized soil in a greenhouse for 15 days for the weed shoot germination test. The weed shoots were cut down prior to being treated every other day with the culture supernatant (crude IAA) at amounts equivalent to 50, 250, and 1250 mg IAA/L. Sterilized distilled water and the IAA production medium were applied as controls. The amount of shoot growth was recorded after 9 days. All plant experiments were performed in accordance with relevant guidelines and regulations.

### Analytical methods

#### Cell dry weight

Yeast cell growth was determined by measuring the optical density at 600 nm (OD_600_) using a spectrophotometer (Genesys 20, Thermo Spectronic, USA). The OD_600_ was converted to cell dry weight (CDW) using a calibration curve CDW (g/L) = OD_600_/1.5057.

#### IAA concentration measurement using HPLC

The culture supernatant was collected by centrifugation for 5 min at 10000 × *g* and analyzed by an HPLC (Agilent Technologies, USA) equipped with a Cosmosil SC18-MS-II column (Nacalai Tesque, Japan) and UV detector (Agilent Technologies, USA) at 280 nm. The mobile phase contained solution A (methanol: acetic acid: water; 10:0.3:89.7 v/v/v) and 60% solution B (methanol: acetic acid: water; 90:0.3:9.7 v/v/v) at a flow rate of 0.3 mL/min as described by Nutaratat et al.^24^. Isocratic elution was used instead of gradient elution. Authentic IAA (Sigma, USA) was used as a standard.

### Statistical analysis

The statistical significance of the results was evaluated by one-way analysis of variance (ANOVA) using IBM SPSS version 16 (SPSS, Cary, USA), and the individual comparisons were evaluated with Duncan’s multiple range test (DMRT). A value of *p* < 0.05 was considered to indicate a significant difference between treatments.

### Ethical approval

This article does not contain any studies with human participants or animals performed by any of the authors.

## Supplementary Information

Supplementary Information.
