# Supplementary material for: Large scale production of indole-3-acetic acid and evaluation of the inhibitory effect of indole-3-acetic acid on weed growth
Source: Sci Rep. 2021 Jun 22;11:13094. doi: 10.1038/s41598-021-92305-w (PMC8219710; doi:10.1038/s41598-021-92305-w)
Supplement: Supplementary file 1 — Supplementary Information. [file 41598_2021_92305_MOESM1_ESM.docx]

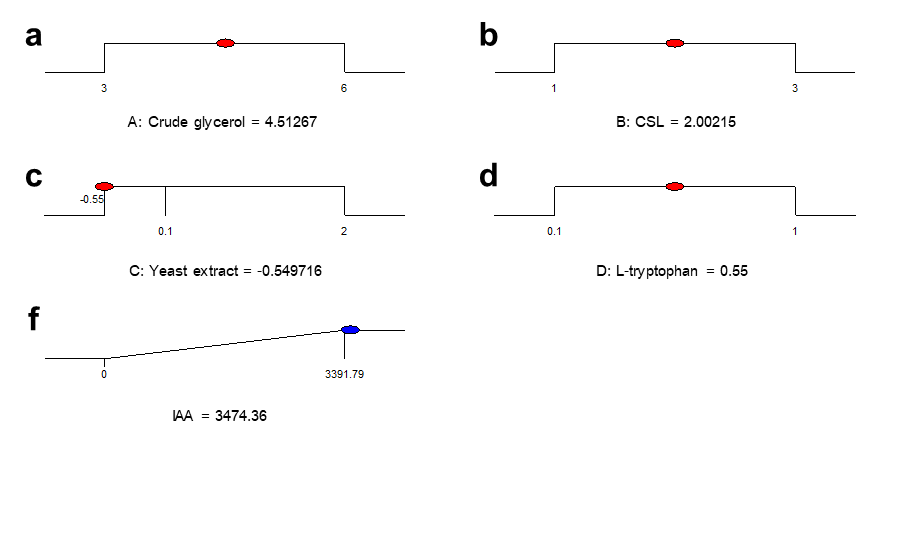


**Figure S1** Summary of the criteria set for the optimization run. The ramps show the predicted levels, and the numbers represent the percentages (%w/v) of (a) crude glycerol, (b) CSL, (c) technical-grade yeast extract, and (d) L-tryptophan.

**Table S1** The variables and their levels for the CCD.

| Factor | Symbol | Coded levels | | | | |
| --- | --- | --- | --- | --- | --- | --- |
|  |  | – α | – 1 | 0 | + 1 | + α |
| Crude glycerol (%w/v) | A | 1.98 | 3 | 4.5 | 6 | 7.02 |
| CSL (%w/v) | B | 0.32 | 1 | 2 | 3 | 3.68 |
| Technical-grade yeast extract (%w/v) | C | – 0.55 | 0.1 | 1.05 | 2 | 2.65 |
| Feed-grade L-tryptophan (%w/v) | D | – 0.21 | 0.1 | 0.55 | 1 | 1.31 |
